# Standardised neonatal parenteral nutrition formulations – an Australasian group consensus 2012

**DOI:** 10.1186/1471-2431-14-48

**Published:** 2014-02-18

**Authors:** Srinivas Bolisetty, David Osborn, John Sinn, Kei Lui

**Affiliations:** 1 Division of Newborn Services, Royal Hospital for Women, Barker Street, Locked Bag 2000, Randwick, 2031 Sydney NSW, Australia; 2 RPA Newborn Care, Royal Prince Alfred Hospital, Sydney, Australia; 3 Department of Neonatology, Royal North Shore Hospital, Sydney, Australia; 4 School of Women’s and Children’s Heath, University of New South Wales, Sydney, Australia; 5 Sydney Medical School, University of Sydney, Sydney, Australia

**Keywords:** Parenteral nutrition, Preterm, Neonates, Standardisation

## Abstract

Standardised parenteral nutrition formulations are routinely used in the neonatal intensive care units in Australia and New Zealand. In 2010, a multidisciplinary group was formed to achieve a consensus on the formulations acceptable to majority of the neonatal intensive care units. Literature review was undertaken for each nutrient and recommendations were developed in a series of meetings held between November 2010 and April 2011. Three standard and 2 optional amino acid/dextrose formulations and one lipid emulsion were agreed by majority participants in the consensus. This has a potential to standardise neonatal parenteral nutrition guidelines, reduce costs and prescription errors.

## Background

Parenteral Nutrition (PN) is routinely used in the neonatal intensive care units (NICUs) in Australia and New Zealand (ANZ). The majority use standardised PN formulations. An email survey found there were 61 different neonatal standardised PN formulations compounded and supplied by one pharmaceutical company in October 2009 [1]. This number does not include formulations prepared in-house in some NICUs. In New South Wales (NSW) alone, there were 32 different amino acid/dextrose (AA/Dextrose) formulations.

In November 2010, representatives from various NICUs in the region joined together to form a Neonatal PN Consensus Group. The main objective was to achieve consensus in developing standardised PN formulations agreeable to the majority of the NICUs and to deliver the recommended parenteral nutrient intakes to the majority of the neonatal population in Perinatal NICUs. Standarised PN formulations are safe, stable and compatible with a long shelf life, are easy and safe to implement so as to avoid errors by rotating frontline staff and are cost effective.

## Consensus process

The group consisted of neonatologists, nursing staff and pharmacists with an input from other experts including a nutritionist and a paediatric gastroenterologist as needed. This group initially consisted of 10 NICUs from the region of New South Wales (NSW) and the Australian Capital Territory (ACT). A series of face-to-face meetings and email correspondence were held between November 2010 and April 2011. A systematic literature search was undertaken and detailed reports were developed for each component of PN. Levels of evidence (LOE) and grades of recommendation (GOR) were allocated according to National Health and Medical Research Council (NHMRC) criteria [2]. Where good published evidence was unavailable, recommendations were discussed and, if necessary, voted upon. Members were given plenty of opportunity and time for a thorough literature review, free and open discussion and an exchange of good practice tips among the units. Each member of the group presented the outcomes of the meetings in their individual units and feedback of the comments and suggestions to the consensus group for their consideration. Representatives from Baxter Pharmaceuticals Australia were present in some of the meetings and correspondence, but their contribution was limited to advice on the feasibility, stability, compatibility and other pharmaceutical related issues on the formulations recommended by the consensus group. All the writings of the manuscript were in no way influenced by the company. The initial consensus process was completed in June 2011. Subsequently, other tertiary NICUs within Australia, New Zealand, Malaysia, Singapore and India joined the consensus group and contributed to the development of consensus formulations. By April 2013, the consensus group consisted of 22 NICUs from Australasia.

## Outcomes

The Consensus Group addressed the following issues.

## Standardised PN versus individualized PN Formulations

Several observational studies have reported that the use of standardised PN solutions is feasible and appears to offer advantages in some but not all clinical settings [3-7]. Individual studies reported use of standardised PN increased energy and amino acid intake [4-6], calcium and phosphate intake [5,6], reduced early weight loss [4] and reduced costs [6]. The single report of significantly better growth, without added clinical or laboratory complications, from use of a more aggressive nutritional approach including individual PN is limited by the 5 year time difference between groups and overall changes in nutritional strategies [7]. The consensus Group agreed that standardised PN offers advantages over routine individualised PN in terms of providing adequate nutrition to the majority of neonates in the NICUs without significant alteration in biochemical responses, with the potential for reduced cost and prescription error (LOE 111–2, GOR C).

### Fluid intake

Systematic review of five studies taken together indicates that restricted water intake significantly increases postnatal weight loss and significantly reduces the risks of patent ductus arteriosus and necrotizing enterocolitis [8]. With restricted water intake, trends were reported toward increased risk of dehydration and reduced risks of bronchopulmonary dysplasia, intracranial haemorrhage, and death, but these trends are not statistically significant. The consensus agreement was new standardised PN should be formulated to provide the Recommended Daily Intakes (RDIs) of nutrients at a total water intake of 150 ml/kg/day. This includes 135 ml/kg/day of AA/Dextrose formulation and 15 ml/kg/day water in the 20% lipid emulsion. There was general agreement on starting parenteral fluid intake at 60 ml/kg/day with daily increase by 20–30 ml/kg/day to an average maximum of 150 ml/kg/day. However, starting fluid intake can be higher in some VLBW infants due to high water loss in the first few days. In such cases, the PN solutions can still be used to provide the necessary daily intakes of nutrients with extra fluid provided as non-PN solutions (LOE 1, GOR B).

### Calorie intake

Recommended parenteral caloric intake varies from 89 to 120 kcal/kg/day in preterm neonates [9-11]. Minimal energy requirements are met with 50–60 kcal/kg/day, but 100–120 kcal/kg/day facilitate maximal protein accretion [12]. A newborn infant receiving PN needs fewer calories (90–100 kcal/kg/day) than a newborn fed enterally because there is no energy lost in the stools and there is less thermogenesis [13]. Trials of early and/or higher energy intake in preterm infants have reported commencing infants on up to 60 kcal/kg/day PN increasing to up to 90–108 kcal/kg/day associated with positive nitrogen balance, glucose and biochemical tolerance, and growth [14-16]. The consensus group proposed standardised starter PN solution at 60 ml/kg/day and lipid emulsion at 1 g/kg/day provides approximately 68 kcal/kg/day, and the standardised preterm PN solution at 135 ml/kg/day and lipid emulsion at 3 g/kg/day provides approximately 100 kcal/kg/day (LOE II, GOR B).

### Amino acids

Trials assessing the efficacy of early (day 1) introduction of amino acids have reported higher blood urea nitrogen levels [17,18] and increased nitrogen/protein accretion [15,17-22] and variable effects on glucose tolerance [15,18,23,24]. Early use of amino acid solutions may improve glucose tolerance [15,18,23] but may not overcome the effect of a high glucose infusion rate [24]. Limited effects on infant growth have been reported from early introduction of amino acid solutions but no improvements in other neonatal outcomes or long term neurodevelopment reported. A systematic review concluded there is insufficient evidence to guide practice regarding the early versus late administration of amino acids to infants less than 37 weeks gestation [25]. The Consensus Group universally agreed to commence parenteral AA within the first 24 hours of birth (LOE I, GOR C).

Trials assessing the efficacy of higher versus lower early amino acid administration also report higher blood urea nitrogen levels [14,18,26], and increased nitrogen/protein accretion [18,27]. Rates of early amino acid intake ranged from 0.5 g/kg/day to 3.5 g/kg/day. The majority of studies did not report any significant differences in initial weight loss or subsequent growth [14,16,18,28]. A single study reported reduced weight loss but increased use of insulin for glucose intolerance from early amino acid and lipid intakes of 2 g/kg/day of each compared to 1 g/kg/day [16]. Data suggest biochemical tolerance and improved nitrogen/protein balance commencing with 2–2.4 g/kg/day amino acid [16,18,26] with limited data reporting the biochemical safety at 3.0-3.5 g/kg/day [15,27]. No improvements in other neonatal outcomes or long term neurodevelopment reported. The Consensus Group agreed to commence parenteral AA at 2 g/kg/day (LOE II, GOR C).

Trials assessing the efficacy of higher maximal rates of amino acid administration have assessed the effects of up to 3.5-4 g/kg/day [14,15,26]. Higher rates of maximal amino acid administration were associated with higher blood urea nitrogen levels [14,26] and increased nitrogen/protein accretion [15]. No significant difference in growth has been reported although this may reflect enteral nutrition practices. Blanco et al. studied the effect of a high parenteral amino acid intake 2 g/kg/d at birth to a max 4 g/kg/d by day 3 in 30 infants born <1000 g and reported biochemical intolerance resulting in discontinuation of intervention in 6 extremely premature infants with peak blood urea nitrogen >21 mmol/L (60 mg/dl) and plasma ammonia level >97 μmol/L [26]. The peak blood urea nitrogen levels were well above normative data [29] although metabolic acidosis was not observed and plasma ammonia levels up to 123 μmol/L may be normal in extremely low birth weight infants [30]. The Consensus Group agreed to incrementally increase amino acid infusions to a maximum 4 g/kg/day over 3–5 days (LOE II, GOR C).

### Carbohydrates

ESPGHAN recommend the rate of glucose infusion should not exceed the maximum rate of glucose oxidation as excessive glucose intake may be responsible for hyperglycaemia [31]. Maximal glucose oxidation has been reported in preterm infants to be 8.3 mg/kg per min (12 g/kg per day) [32,33], and in term infants 13 mg/kg per min (18 g/kg per day) [34,35]. Elevated neonatal blood glucose concentration has been linked to adverse outcomes including death [36,37], intraventricular haemorrhage [36], late onset bacterial infection [38], fungal infection [39,40], retinopathy of prematurity [41-43] and necrotizing enterocolitis [38]. Attempts to maintain glucose intake using insulin have yielded variable results [44,45]. For prevention of hyperglycaemia, a systematic review found two small trials which compared lower versus higher glucose infusion rates [45]. These trials provided some evidence that a lower glucose infusion rate reduced mean blood glucose concentrations and the risk of hyperglycaemia, but had insufficient power to test for significant effects on death or major morbidities [16,46]. The trials had glucose infusion rates up to 8.4 mg/kg/min in the first 7 days in the highest infusion group. In contrast, the multicentre trial of insulin infusion reported that although insulin infusion reduced mean glucose concentrations and reduced hyperglycaemia, it resulted in an increase in the risk of death before 28 days and an increase in the proportion of neonates with a hypoglycaemic episode [47]. Glucose infusion rates were higher in the insulin group compared to the standard care group (median 9.3 versus 7.6 mg/kg/min). The review concluded the evidence does not support the routine use of insulin infusions to prevent hyperglycaemia in VLBW neonates. A second systematic review found two trials that compared use of insulin infusion for treatment of hyperglycaemia [44]. Collins 1991 reported that infants treated with insulin infusion received significantly higher glucose infusion rates than infants treated with no glucose reduction (20.1 ± 2.5 versus 13.2 ± 3.2 mg/kg/min), and significant increases in non-protein energy intake and short-term weight gain [48]. Meetze 1998 reported glucose infusion rate averaged approximately 10.0 mg/kg/min in the insulin infusion group and approximately 7.6 mg/kg/min in the glucose reduction group, and significant increase in total energy intake [49]. Neither reported a significant difference in neonatal mortality or morbidity.

Proposed standard preterm and term PN formulations contain 10% and 12% dextrose respectively, providing 13.5 (9.4 mg/kg/min) and 17 g/kg/day (11.8 mg/kg/min) at 135 ml/kg/day respectively (LOE 1, GOR C).

### Sodium, Potassium and Chloride

Higher early sodium intake may be associated with early hypernatraemia and increased oxygen requirements to 28 days [50-53]. There is insufficient evidence to determine an effect on other neonatal outcomes and mortality [50,51]. Subsequent higher sodium intake may reduce the incidence of hyponatraemia [50-52,54]. The consensus group agreed on minimal sodium intake approximately 1 mmol/kg/d on day 1 using a starter PN formulation, with an increase to a maximum 4.5 mmol/kg/d in preterm and 3.5 mmol/kg/day in term infants (LOE II, GOR C).

Hyperkalaemia is a common complication in the first 48 hours of life in very low birth weight and/or very preterm [55]. In a clinical trial, it was not affected by early and high administration of protein [26]. After 3 days, balance studies reported a potassium intake of 2–3 mmol/L/day resulted in a net retention of 1–2 mmol/day [56,57]. The consensus group agreed on minimal potassium intake using starter PN formulation, with an increase in standard formulations to a maximum 3.0 mmol/kg/d in preterm and 3.5 mmol/kg/day in term infants (LOE III-2, GOR C).

Hyperchloraemia (>115 mmol/L) is a common problem in VLBW infants on PN and is associated with acidosis [58,59]. Trial evidence found the incidence of hyperchloraemia and acidosis is reduced by partly replacing chloride with acetate in parenteral nutrition [58]. Consensus was to adopt the trial recommendation of the first 3 mmol/kg/day of anion to be provided as chloride, next 6 mmol/kg/day of anion to be provided as acetate and thereafter as chloride again. Supplementation of acetate beyond this level was associated with hypercarbia [58] (LOE II, GOR C).

### Calcium, Phosphate and Magnesium

The range of recommended doses of Ca and P delivered by PN in preterm infants is wide, varying from 1.3-3 mmol Ca/kg/day and 1.0-2.3 mmol P/kg/day, with a Ca:P ratio in the range of 1.3-1.7 [9,10]. Lower doses may be inadequate for some infants, compromising short and long-term bone formation. Trials have reported higher intakes of calcium and phosphate increase mineral retention, bone mineral content and bone strength [60,61]. However, the threshold for calcium-phosphate precipitation limits the delivery of appropriate amounts of Ca and P by PN [62]. Substitution of inorganic phosphate by organic phosphate improves physicochemical compatibility which trial evidence reported allowed for increased mineral intake and mineral retention [63]. However, there is no registered organic phosphate in Australia. The consensus formulations are not to exceed 12 mmol/L of calcium and 10 mmol/L of inorganic phosphorus due to concerns about physicochemical compatibility and precipitates (LOE II, GOR C).

Balance studies indicate that a magnesium intake 0.375 mmol/kg/day may result in elevated serum magnesium levels without clinical evidence of hypomagnesaemia, and for Mg intake a minimum of 0.2 mmol/kg/day and 0.3 mmol/kg/day would be appropriate for LBW infants [64,65] (LOE 111–3, GOR C).

### Heparin

Systematic review of prophylactic use of heparin for peripherally placed percutaneous central venous catheters found a reduced risk of catheter occlusion with no statistically significant difference in the duration of catheter patency, risk of thrombosis, catheter related sepsis or extension of intraventricular haemorrhage [66]. Heparin was added at 0.5 to 1 IU/ml to parenteral nutrition solution with no adverse effect reported. The consensus formulations contain heparin 0.5 IU/ml (LOE I, GOR C).

## Consensus AA/dextrose formulations

Based on the above principles, the Consensus Group agreed on 3 standard and 3 optional standardised AA/Dextrose formulations (Table 1). Three Standard formulations are as follows:

(1) Starter PN – suitable for both term and preterm neonates in the first 1–2 days. It contains 33 g/L of amino acids (AA 3.3%) and 10% dextrose. It provides 2 g/kg/day of amino acids at 60 ml/kg/day. It contains minimum Na (15 mmol/L) and no potassium. This formulation may be used up to 120 ml/kg/day for occasional infants on fluid restriction and renal impairment. At 90 ml/kg/day this solution provides 3 g/kg/day amino acids beyond which the recommended protein intake is exceeded for term infants [10]. At 120 ml/kg/day the solution provides 4 g/kg/day of amino acids beyond which the recommended preterm intake is exceeded [10].

(2) Standard Preterm PN – This is suitable for stable preterm infants after the first 24 hours. It provides 4 g/kg/day of amino acids and 12 g/kg/day of glucose at 135 ml/kg/day.

(3) Standard Term PN – Suitable for stable term neonates after the first 24 hours of life. It provides 3 g/kg/day of amino acid and 18 g/kg/day of glucose at 135 ml/kg/day.

**Table 1:** Consensus AA/dextrose formulations

	**Starter PN**	**Standard preterm PN**	**High sodium preterm PN**	**7.5% dextrose preterm PN**	**Term PN**
**Conc/Litre**					
AA, g	33	30	30	30	23
Glucose, g	100	100	100	75	120
Na, mmol	15	33	60	33	25
K, mmol	0	22	22	22	20
Cl, mmol	12	16	36	22	26
Ca, mmol	12	12	12	12	12
Mg, mmol	1.5	1.5	1.5	1.5	1.5
Ph, mmol	10	10	10	10	10
Acetate, mmol	5	40	44	40	13.5
Zinc, μg	0	3260	3260	3260	1900
Selenium, μg	0	20	20	20	20
Iodine, μg	0	8	8	8	8
Heparin, units	500	500	500	500	500
Osmol, mosm/L	813	790	790	651	847
**At 135 ml/kg/Day**					
Energy, Kcal/kg/day†	72	70	70	57	77
AA, g/kg/day	4.5	4	4	4	3
Glucose, g/kg/day	13.5	13.5	13.5	10	16.2
Na, mmol/kg/day	2	4.5	8.1	4.5	3.4
K, mmol/kg/day	0	3	3	3	2.7
Cl, mmol/kg/day	1.6	2.2	4.9	3	3.5
Acetate mmol/kg/day	0.7	5.4	6	5.4	1.8
Ca, mmol/kg/day	1.6	1.6	1.6	1.6	1.6
Ph, mmol/kg/day	1.4	1.4	1.4	1.4	1.4
Mg, mmol/kg/day	0.2	0.2	0.2	0.2	0.2
Zinc, μg/kg/day	0	440	440	440	256
Selenium, μg/kg/day	0	2.7	2.7	2.7	2.7
Iodine, μg/kg/day	0	1	1	1	1

†Energy rates are based on estimated 4 kcal per each gram of glucose and protein.

Two optional AA/Dextrose solutions have also been agreed upon by majority units to provide the PN for preterm infants with hyponatraemia and hyperglycaemia.

(1) High Sodium Preterm PN – similar to standard preterm PN with higher Na content (60 mmol/L) provides Na 8 mmol/kg/day at 135 ml/kg/day along with increased chloride and acetate.

(2) 7.5% Dextrose Preterm PN – similar to standard preterm TPN with the exception of 7.5% dextrose providing 10 g/kg/day glucose at 135 ml/kg/day.

### Lipids

Administration of lipid in premature infants requiring PN provides essential fatty acids and increases caloric intake with a low volume [10]. Two systematic reviews found that although no side effects were reported there was no statistically significant benefit of introducing lipids before two to five days of age, including no beneficial effects on growth [67,68]. However, essential fatty acid deficiency occurs rapidly and can be prevented with introduction of as little as 0.5 to 1 g/kg/day of lipid infusion [67]. The Consensus agreed timing of commencement of parenteral lipid will be determined by individual NICUs with day 1 administration as an option (LOE 1, GOR C).

Several types of intravenous lipid emulsions (IVLE) are available for neonatal use including 100% soybean oil based IVLEs (e.g. Intralipid 20%, Ivelip 20%); mixed 80% olive oil/20% soybean oil IVLE (e.g. Clinoleic 20%); mixed 30% soybean oil/25% olive oil/30% medium-chain triglyceride oil/15% fish oil IVLE (e.g. SMOFlipid); and 100% fish oil based IVLE (e.g. Omegaven). The IVLEs are largely well tolerated. No reproducible clinical benefits have been reported for any specific IVLE in newborn infants [69-80]. Although reduced peroxide formation [81] and some biochemical difference in infants have been reported [78], a reduced incidence of cholestasis has not been reported using any specific preparation in newborns to date. After consideration of costs, the consensus group agreed to continue with the current practice of olive oil based emulsion in the majority of NICUs (LOE II, GOR C).

The current Consensus emulsion contains 20% lipid and 80% water (Clinoleic 20%). In view of benefits in neonatal morbidities reported in a systematic review [8], the group proposed to include lipids in the total fluid intake, which equates to 15 ml/kg/d of water when the lipid intake reaches 3 g/kg/day (LOE I, GOR B). The proposed lipid emulsion with the vitamins are available in 2 volumes: 50 ml opaque syringes and 150 ml bags. Each 50 ml contains the following: Clinoleic 20% - 36 ml, Vitalipid 10% - 11.2 ml and Soluvit reconstituted in water for injection – 2.8 ml.

### Vitamins

Water and fat soluble vitamins (Soluvit and Vitalipid 10%) are added to the lipid emulsion to increase the vitamin stability [82]. Table 2 shows the amount of vitamins supplied to infants through the proposed lipid emulsion when run at 3 g/kg/day. The doses of vitamin K, pyridoxine and riboflavin are above recommended parenteral doses, and ascorbate below [9,10]. Loss of vitamins and formation of peroxides from exposure to light is substantially reduced by adding the preparation to the lipid infusate, covering and use of amber/dark syringes and tubing [83-85] (LOE II, GOR B).

**Table 2:** Parenteral RDIs of vitamins and the comparison to the consensus formulations

**Element**	**Parenteral RDIs†**	**Lipid emulsion @ 3 g/kg/day**
Vit A, IU/kg/day	700-1500	920
Vit D, IU/kg/day	40-160	160
Vit E, IU/kg/day	2.8-3.5	2.8
Vit K, μg/kg/day	10	80
Ascorbate, mg/kg/day	15-25	10
Thiamine, μg/kg/day	200-500	310
Riboflavine, μg/kg/day	150-200	360
Pyridoxine, μg/kg/day	150-200	400
Nicotinamide, mg/kg/day	4-6.8	4
Pantothenate, mg/kg/day	1-2	1.5
Biotin, μg/kg/day	5-8	6
Folate, μg/kg/day	56	40
Vit B12, μg/kg/day	0.3	0.5

†RDIs adapted from AAP 2009 and ESPGHAN2005 recommendations.

Optimal doses and conditions of infusion for vitamins in infants and children have not been established [10]. The doses of many vitamins (eg Thiamine, Riboflavin, Folate, Vitamin B12, Pyridoxine, and Vitamin C) are largely determined by studies determining vitamin levels during intravenous supply undertaken with commercially available mixtures [10,86-88].

Vitamin A: Systematic review found that supplementation of very low birthweight infants with vitamin A is associated with reduction in death or oxygen use at one month of age and oxygen use at 36 weeks’ postmenstrual age, but this needs to be balanced against the lack of other proven benefits and the acceptability of treatment [89]. Current dosing recommendations for parenteral vitamin A supplementation for premature infants are based on clinical studies measuring vitamin levels during supplementation [10] (LOE I GOR C).

Vitamin C: A single randomised trial reported no significant benefits or harmful effects were associated with treatment allocation to higher or lower ascorbic acid supplementation throughout the first 28 days [90]. The lower group received 10 mg parenterally provided in Soluvit and Vitalipid (LOE II, GOR C).

Vitamin D: The consensus formulation delivers vitamin D 160 IU/kg/day, above the minimal required vitamin D intake reported to maintain 25(OH) vitamin D levels [64] and consistent with studies reporting stable vitamin D status in preterm infants on PN [91] (LOE III-3, GOR C).

Vitamin E: Systematic review found Vitamin E supplementation in preterm infants reduced the risk of intracranial hemorrhage but increased the risk of sepsis. It concluded evidence does not support the routine use of vitamin E supplementation by intravenous route at high doses or aiming at serum tocopherol levels greater than 3.5 mg/dL, supporting the current recommendation for parenteral intake of vitamin E [92] (LOE I, GOR B).

Vitamin K: Preterm infants who received intramuscular Vitamin K 1 mg at birth, followed by parenteral intake (60 μg/day for infants <1000 g and 130 μg/day for infants 1000 to 3000 g) had much higher vitamin K plasma concentrations at 2 and 6 weeks of age than previously reported in healthy, term, formula-fed infants (4–6 ng/mL) [93]. The only formulation available in Australia delivers in excess of current recommendation [10] and is associated with high vitamin K plasma concentrations (LOE 111–3, GOR C).

### Trace elements

Chromium, copper, iodine, manganese, molybdenum, selenium and zinc are essential micronutrients involved in many metabolic processes. Table 3 shows the parenteral RDIs of trace elements (EPSGHAN) [10] and the comparison to the consensus group formulations. Nutritional deficiency in low birth weight or preterm infants on parenteral nutrition has been mostly reported for zinc and copper [94]. The risk is substantially increased in surgical infants with increased gastrointestinal losses. There are no reports of clinical manganese deficiency in newborns on PN [94]. Copper and manganese may need to be withheld if the neonate develops PN-associated liver disease. Copper has the potential for hepatotoxicity and biliary excretion is important for manganese which is potentially neurotoxic [94]. Low blood selenium concentrations in preterm infants have been reported as a potential risk factor for chronic neonatal lung disease and retinopathy of prematurity [95]. Iodine deficiency and excess have been reported in preterm infants, with iodine excess associated with transient hypothyroidism [96-98]. There have been few reports of chromium deficiency in humans [94]. PN solutions may be contaminated with chromium, causing serum concentrations to be significantly higher (10%-100%) than recommended [99]. There is a concern excess chromium intake may be associated with renal impairment in preterm infants [100].

**Table 3:** Parenteral RDIs of trace elements and the comparison to the consensus formulations

**Element**	**Parenteral RDIs†**	**PN solution@ 135 ml/kg/day**
Zinc, μg/kg/day	450–500 (preterm)	440 (preterm)
	250 (term)	256 (term)
Selenium, μg/kg/day	2-3	2.7
Iodine, μg/kg/day	1	1
Chromium, μg/kg/day	0	0 (may ‘contaminate’)
Copper, μg/kg/day	20	0 initial
		20*
Manganese μg/kg/day	1	0 initial
		1*
Molybdenum μg/kg/day	1	0 initial
		1*

†RDIs adapted from ESPGHAN2005 recommendations. *Consensus recommendation to add if more than 2 to 4 weeks PN.

Zinc: Zinc doses are derived from clinical trials reporting zinc levels and zinc balance in preterm and term infants [101-103]. Clinical benefits from different parenteral intake have not been reported in trials. Parenteral zinc is recommended at a dose of 450–500 μg/kg/day for premature infants and 250 μg/kg/day for infants less than 3 months [10]. Zinc is recommended to be added to solutions of patients on short-term PN from commencement [10,94] (LOE II. GOR C).

Copper: Copper doses are derived from clinical trials reporting copper levels and copper balance in preterm and term infants [101-103]. Clinical benefits from different parenteral intake have not been reported in trials. Parenteral copper intake is recommended at a dose of 20 μg/kg/day [10], and commenced 2 to 4 weeks after PN commencement [94]. Copper should be carefully monitored in patients with cholestatic liver disease [10,94] (LOE II, GOR C).

Selenium: Systematic review found supplementing very preterm infants with selenium is associated with a reduction in episodes of sepsis, but was not associated with improved survival, a reduction in neonatal chronic lung disease or retinopathy of prematurity. Doses of 3 μg/kg/d may prevent a decline in cord levels and doses up to 7 μg may be required to achieve concentrations above those in cords and close to concentrations found in healthy breast fed infants [95]. Selenium supply of 2 to 3 μg/kg/day is currently recommended for parenterally fed LBW infants [10] (LOE I, GOR C).

Iodine: Observation data suggest preterm infants receiving PN containing a mean iodine intake of 3 μg/kg/day are in negative iodine balance [97]. However, the relationship to transient thyroid dysfunction in preterm infants is unclear. Enterally fed infants are recommended to receive iodine 11–55 μg/kg/day [104], although a small trial reported no evidence of an effect of higher enteral iodine intake on thyroid hormone levels in very preterm infants [105]. Iodine intake needs to be appraised in the context of iodine status and iodine exposures of pregnant women and their infants. The recommended parenteral intake is currently 1 μg/kg/day [10] (LOE III-3, GOR D).

Manganese: A randomised trial comparing PN intake of 1 μmol/kg/day versus 0.0182 μmol/kg/day reported no significant difference between peak manganese levels between groups [106]. However, peak levels in both groups were above normal ranges and there was no significant difference between groups in incidence of cholestasis or morbidity and mortality. Subgroup analysis raised the concern that infants on the higher dose for >14 days had an increased rate of cholestasis. Supplementation should be stopped in infants with cholestasis [94]. In infants receiving long-term PN, a low dose supply of no more than 1 μg/kg/day (0.0182 μmol/kg/day) is recommended [10] (LOE II, GOR C).

Molybdenum: Deficiency has not been reported in newborns. Observational data led to the speculation that an intravenous intake of 1 μg/kg/day would be adequate for the LBW infant [107]. Intravenous molybdenum supply of 1 μg/kg/day (0.01 μmol/kg/day) is recommended for the LBW infant [10] (LOE III-3. GOR D).

There are 2 commercial trace element formulas available in Australia and neither of them has the right mix of trace elements for neonatal use. AUSPEN Neonatal Trace elements (Baxter Healthcare Pty Ltd) contains more copper and manganese but less zinc. Peditrace (Fresenius-Kabi Pty Ltd) contains fluorine. Consensus was to add zinc, selenium and iodine as individual trace elements to all AA/Dextrose formulations. Exception is the starter formulation to which trace elements could not be added due to physico-chemical compatibility concerns. For those infants, who are on exclusive PN for more than 2–4 weeks with minimal enteral intake, other trace elements (copper, manganese and molybdenum) can be added to the formulations.

### Duration of infusion

Parenteral nutrition solution: In a randomised trial enrolling 166 infants, there is no significant difference in bacterial or fungal colonisation of infusate or neonatal sepsis in infants receiving 24 or 48 hour infusions of parenteral nutrition solution [108]. A before-after intervention study reported extending PN solution hang time from 24 to 48 hours did not alter central line associated blood stream infection rate and was associated with a reduced PN-related cost and perceived nursing workload [109].

Lipid infusion: In previously mentioned randomised trial, fungal contamination may be increased in infants receiving lipid infusion for 24 hours compared to 48 hours [108]. In another trial randomising PN set changes (rather than infants), microbial contamination of infusion sets was significantly more frequent with 72-hour than with 24-hour set changes in neonates receiving lipid solutions [110].

### Physico-chemical stability

Bouchoud and colleagues studied the long term physico-chemical stability of the standardised AA/Dextrose and lipid emulsions [82]. The formulations tested were of similar concentrations to the ones we proposed in our consensus. Their AA/Dextrose solutions contain 3% AA, 10.8% glucose and heparin 0.5 IU/ml and the lipid emulsion was lipofundin with vitamins added to it. AA/Dextrose was supplied in multilayered plastic bag and lipid emulsions were supplied in polypropelene syringes, similar to our practice. They demonstrated an optimal physical and chemical stability of AA/Dextrose solutions even if they are stored for a few weeks at room temperature. Similarly, lipid emulsions containing vitamins showed negligible difference in physical stability, lipid peroxide and vitamin levels when stored at room temperature for a few weeks.

The consensus recommended a hang time of 48 hours for PN solution and lipid (LOE II, GOR C).

## Discussion

To our knowledge, this is the first consensus on the standardisation of parenteral nutrition across a majority of units. The main consensus achievement was to develop evidence based standardised formulations which can be implemented with minimal need for individualisation in the newborn population, and to provide framework for further development as new evidence becomes available. Success was facilitated by the majority NICUs participating in the consensus group already using standardised PN formulations. At the time of writing up this manuscript, 20 NICUs from Australia, New Zealand, Singapore and Malaysia are currently participating in the consensus group. NICUs in the group variably implemented some or all the formulations.

The consensus group negotiated a universal price for the whole Australia and fixed for 3 years. A common PN clinical protocol has been developed which is being implemented across many NICUs.

There are some limitations and gaps in the formulations and some unresolved issues in the initial consensus. Starter AA/dextrose formulation contain 33 g/L of AA, which provides 2 g/kg/d of AA at 60 ml/kg/d. However, there are some extreme preterm infants who may be receiving other intravenous infusions which clinicians include in the total fluid intake resulting in a reduced calorie and AA intake. Pharmaceutical advice at the time of the consensus was not to exceed 33 g/L of AA due to stability and compatibility concerns. There was consensus on the addition of zinc, selenium and iodine but we were unable to reach any consensus on the routine addition of other trace elements.

Currently, there is an observational study underway testing the safety and efficacy of the new consensus formulations. The findings of this study may help further improve these formulations.

## Conclusion

In conclusion, consensus is achievable in standardising the PN formulations in the neonatal intensive care units. This has the potential to improve nutrient intakes, quality control, cost effectiveness and reduce prescription errors.

## Abbreviations

AA: Amino acids; ACT: Australian capital territory; ANZ: Australia and New Zealand; ESPGHAN: European society of paediatric gastroenterology, hepatology and nutrition; GOR: Grade of recommendation; LOE: Level of evidence; NICU: Neonatal intensive care unit; PN: Parenteral nutrition; RDI: Recommended daily intakes; VLBW: Very low birthweight.

## Competing interests

Authors have no competing (financial or non-financial) interests to declare.

## Authors’ contribution

SB was a core group member of the consensus group and conceptualized the consensus process and along with co-authors organised all the proceedings of the meetings, and drafted the initial manuscript. DO was a core group member of the consensus group, contributed to the concept and design of the consensus, and performed critical review of the level of evidence and grading of recommendation, JS was a core group member of the group, contributed to the concept and design of the consensus and contribution to the writings of all proceedings, KL was a core group member and contributed to the concept, design and networking for the consensus, reviewed and assisted in writing up manuscript. All authors approved the final manuscript as submitted.

## Members of the Consensus Group in alphabetical order

Centenary Hospital, Canberra, Australia – Dr Alison Kent

Children’s Hospital at Westmead, Australia –Dr Amit Trivedi, Ms Demiana Yaacoub

Flinders Medical Centre, Adelaide, Australia – Dr Scott Morris, Dr Peter Marshall

Gold Coast Hospital, Gold Coast, Australia – Dr Pita Birch

Hawkes Bay Hospital, Hastings, New Zealand – Dr Jenny Corban

Hospital Putrajaya, Malaysia – Dr Vidya Natthondan

Hospital Sg Buloh, Malaysia – Dr See Kwee Ching

John Hunter Children’s Hospital, Newcastle, Australia – Dr Chris Wake

KEM Hospital, Pune, India – Dr Umesh Vaidya

Liverpool Hospital, Liverpool, Australia – Dr Rodney Tobiansky, Ms Natalie Pazanin

Monash Medical Centre, Melbourne, Australia – Dr Kenneth Tan

Nepean Hospital, Nepean, Australia – Dr Lyn Downe, Dr Girish Deshpande

Royal North Shore Hospital, St Leonards, Australia – Dr John Sinn

Royal Prince Alfred Hospital, Camperdown, Australia – Dr David Osborn

Royal Hobart Hospital, Hobart, Australia – Dr Tony De Paoli

Royal Hospital for Women, Randwick, Australia – Dr Srinivas Bolisetty, Assoc Prof Kei Lui

St John of God Hospital, Subiaco, Australia – Dr Joanne Colvin

Sydney Children’s Hospital, Randwick, Australia – Dr Hari Ravindranathan, Dr Nitin Gupta, Mr Declan Gibney

Westmead Hospital, Westmead, Australia – Dr Melissa Luig, Mr Kingsley Ng, Ms Tamara Pham

Women’s and Children’s Hospital, Adelaide, Australia – Dr Andrew McPhee

## Pre-publication history

The pre-publication history for this paper can be accessed here:

http://www.biomedcentral.com/1471-2431/14/48/prepub
